# Overcrowding analysis in emergency department through indexes: a single center study

**DOI:** 10.1186/s12873-022-00735-0

**Published:** 2022-11-18

**Authors:** Ylenia Colella, Danilo Di Laura, Anna Borrelli, Maria Triassi, Francesco Amato, Giovanni Improta

**Affiliations:** 1 Department of Electrical Engineering and Information Technologies, University of Naples “Federico II”, Naples, Italy; 2 “San Giovanni Di Dio E Ruggi d’Aragona” University Hospital, Salerno, Italy; 3 Department of Public Health, University of Naples “Federico II”, Naples, Italy; 4 Interdepartmental center for research in healthcare management and innovation in healthcare (CIRMIS), University of Naples “Federico II”, Naples, Italy

**Keywords:** Emergency Department, Overcrowding, EDWIN Index, NEDOCS Index

## Abstract

**Introduction:**

Overcrowding in the Emergency Department (ED) is one of the major issues that must be addressed in order to improve the services provided in emergency circumstances and to optimize their quality. As a result, in order to help the patients and professionals engaged, hospital organizations must implement remedial and preventative measures. Overcrowding has a number of consequences, including inadequate treatment and longer hospital stays; as a result, mortality and the average duration of stay in critical care units both rise. In the literature, a number of indicators have been used to measure ED congestion. EDWIN, NEDOCS and READI scales are considered the most efficient ones, each of which is based on different parameters regarding the patient management in the ED.

**Methods:**

In this work, EDWIN Index and NEDOCS Index have been calculated every hour for a month period from February 9th to March 9th, 2020 and for a month period from March 10th to April 9th, 2020. The choice of the period is related to the date of the establishment of the lockdown in Italy due to the spread of Coronavirus; in fact on 9 March 2020 the Italian government issued the first decree regarding the urgent provisions in relation to the COVID-19 emergency. Besides, the Pearson correlation coefficient has been used to evaluate how much the EDWIN and NEDOCS indexes are linearly dependent.

**Results:**

EDWIN index follows a trend consistent with the situation of the first lockdown period in Italy, defined by extreme limitations imposed by Covid-19 pandemic. The 8:00–20:00 time frame was the most congested, with peak values between 8:00 and 12:00. on the contrary, in NEDOCS index doesn’t show a trend similar to the EDWIN one, resulting less reliable. The Pearson correlation coefficient between the two scales is 0,317.

**Conclusion:**

In this study, the EDWIN Index and the NEDOCS Index were compared and correlated in order to assess their efficacy, applying them to the case study of the Emergency Department of “San Giovanni di Dio e Ruggi d’Aragona” University Hospital during the Covid-19 pandemic. The EDWIN scale turned out to be the most realistic model in relation to the actual crowding of the ED subject of our study. Besides, the two scales didn’t show a significant correlation value.

## Introduction

Overcrowding in emergency departments (EDs) is one of the key challenges in effective hospital administration. Overcrowding in hospital context is described as "a condition in which the identified need for emergency care exceeds available resources in the ED", according to The American College of Emergency Physicians (ACEP) Crowding Resources Task Force. When there are more patients than staffed ED treatment beds and wait times surpass a tolerable length, this scenario arises in hospital EDs.

In many nations, overcrowding in emergency departments is a concern, with major ramifications for patient satisfaction, staff productivity, and the system as a whole, as well as it represents an increase in expenses [1].

Two aspects of the hospital system that might be crucial, according to Gurol-Urganci et al. [2], are demand fluctuation and lead-time variability. In fact, one of the most common reasons of ED overcrowding is the delay in sending patients to hospital surgical units after they have been registered and assessed, resulting in individuals waiting in the ED. Other factors that may contribute to ED overcrowding include the expanding senior population, the increased number of difficult cases, and patients with comorbidities [3–5].

ED congestion is also related to a variety of negative outcomes, including longer treatment durations, preventable medical errors, and the proportion of patients who leave the ED without receiving a medical evaluation from a healthcare professional [6].

Many scientific studies from various fields of study have recently addressed the problem of improving the quality of healthcare services through the use of managerial, statistical, and modeling tools to address issues such as prolonged hospital stays, increased waiting times, appointment scheduling, and other issues[7–20].

Several strategies have been used to increase the efficiency of procedures, healthcare processes in various hospitals, and logistics and resource management; ranging from Lean and Six Sigma to simulation [7, 8, 15, 21], many techniques have been used to address the problem of overcrowding in EDs [22–24].

A considerable number of scientific studies have addressed this subject in recent years, including contributions from several fields of research [25, 26]. The fundamental issue is that there is no one standard metric of hospital performance, hence there is no global standard definition of congestion in emergency departments. The United Kingdom was the first country to require a few clinical indicators at the national level in 1990 [27]. In 1996, the Department of Health issued recommendations stating that a patient must be seen within five minutes of arriving at the hospital [28]. The Department of Health assessed and compared first-aid performance, using rapidity in diagnosing the patient's condition as a criterion [29]. It implemented the "4-h rule" in 2004, requiring that 98 percent of patients be examined and either hospitalized or released within four hours after arriving at the emergency room. Several other clinical indicators were created in the years that followed. However, Jones and Schimanski [30] shown in 2010 that the implementation of an ED time objective and the accompanying huge financial commitment in the United Kingdom did not result in a consistent improvement. As a result, the authors cautioned countries interested in replicating the United Kingdom's experience. A year later, the Minister of Health issued a message to all NHS executives on the Department of Health (DH) website, announcing the repeal of the 4-h regulation as of April 2011 [31]. In December 2010, a new set of indexes for evaluating the performance of EDs was revealed; these indexes were first applied in April 2011 [27].

There are many indexes of ED crowding identified in the scientific literature; below we list the main ones:

- four multidimensional indexes: EDWIN [Emergency Department Work Index] [32], READI [Real-time Emergency Analysis of Demand Indicators] [33], NEDOCS [National ED Overcrowding Study Index] [38] and NEAT [National Emergency Access Target] [34]; these scales, EDWIN and NEDOCS in particular, have shown a high capacity to reflect the current level of overcrowding in the ED. The EDWIN score is related to the ESI (Emergency Severity Index) which determines priority levels in correlation with clinical conditions to the need for resources; indeed NEDOCS score is based on parameters of institutional structures and on activity variables so it evaluate different aspects of patient management in the ED.
- five input indexes: total capacity of first aid, number of patient arrivals in six hours, ambulance transport number, number of patients waiting for medical treatment, and number of patients in the waiting room;
- three throughput indexes: length of stay in the emergency department [ED LOS], wait time for a first appointment, and time spent in waiting room;
- two output indexes: number of patients in the emergency room and percentage of total beds occupied.

Following the definition of the indexes, several investigations were done to validate them. To quantify the effect of crowding on patient satisfaction, Tekwani et al. [35] performed a survey on a sample of patients released from the emergency room after an eight-month delay. The degree of crowding in an ED was measured by the NEDOCS index before and after the introduction of a new management tools in the administration of hospital beds in a research by Todisco [36].

Several studies have examined the EDWIN and NEDOCS indexes' performance in assessing overcrowding. In 2006, Weiss et al. [37] questioned whether the NEDOCS and EDWIN indexes are equally sensitive and specific for the problem of overcrowding. The authors proved that both indexes, particularly the NEDOCS index, have high accuracy for forecasting emergency department overcrowding on a sample of 130 patients in that research. Bernstein et al. [32] compared the results of the EDWIN index to the results of doctor and nurse perceptions of emergency department crowding. The EDWIN index and the staff's crowding evaluation were shown to have a good association in the study.

Despite the fact that the EDWIN and NEDOCS indexes were developed using distinct approaches, they both seek to capture the comparable result value of real-time specialists' opinions on ED crowding and are thus useful estimate tools.

The aim of this work is to analyze the NEDOCS and EDWIN values for ED of the "San Giovanni di Dio e Ruggi d'Aragona" University Hospital (Salerno, Italy) and to evaluate their effectiveness. In particular, this work is an extension of the short paper presented at the BECB Conference in August, 2021 (2021 International Symposium on Biomedical Engineering and Computational Biology) [38]. More in detail, in the work presented at the BECB 2021 Conference, an analysis of the overcrowding indices was carried out by comparing the results obtained from the processing of the data of the same seven day period in two different years, 2020 and 2021, pre and post Covid-19 pandemic, respectively*. The aim was to highlight the different degree of overcrowding due to the different way of perceiving the need to access the hospital by the population after one year of pandemic. In order to study the begin of this trend*, in this paper the overcrowding indicators have been registered every hour over two months periods, from February 9th to March 9th, 2020 and from March 10th to April 9th, 2020. In this way we want to better assess the direct impact of Covid-19 on ED overcrowding thanks to the analysis that took place over two months. Besides, differently from the work presented at the BECB 2021 Conference, another additional point that that gives greater texture to this study is the correlation analysis carried out between NEDOCS and EDWIN.

## Methods

### Data collection

This is a prospective study of the evaluation techniques of ED overcrowding applied on the case study of the Emergency Department of “San Giovanni di Dio e Ruggi d’Aragona” University Hospital. The NEDOCS and the EDWIN indexes have been calculated every hour for a month period from February 9th to March 9th, 2020 and for a month period from March 10th to April 9th, 2020. The choice of the period is *strictly* related to the date of the establishment of the lockdown in Italy due to the spread of Coronavirus; in fact on 9 March 2020 the Italian government issued the first decree regarding the urgent provisions for upgrading of the National Health Service in relation to the COVID-19 emergency [39]. *For this reason, in order to better understand the trend of the phenomenon that involved the ED departments we choose to study overcrowding indexes during a month before and a month after the start of the pandemic emergency in Italy.*

All values for the EDWIN and NEDOCS models have been calculated using data available for download form the hospital’s triage system database in order to not involve any patient contact.

### The EDWIN model

The EDWIN index (Emergency Department Work Index) is defined as [35]

$$\frac{\sum_{i}{n}_{i}*{t}_{i}}{{N}_{a}*({B}_{T}-{B}_{A})}$$

where n_i_ is the number of patients in the emergency room in the i-th triage category, t_i_ is the triage category (scale of 1 to 4, where 4 is the gravest), N_a_ is the number of physicians on duty, B_T_ is the number of treatment beds and B_A_ is the number of patients in the ED.

In this study has been assigned a number to the patients in the ED based on the corresponding category of triage; t_i_ is 1 for patients with the white code, 2 for patients with the green code, 3 for patients with the yellow code and 4 for patients with the red code. The number of treatment beds located in the ED is 25 while the number of attending doctors is 3.

The number of patients for each triage category for each day of the period considered for the study is shown in Tables 1 and 2.

**Table 1:** Number of patients for triage category from February 9th to March 9th, 2020

Data	#patients in white category of triage	#patients in green category of triage	#patients in yellow category of triage	#patients in red category of triage
09/02	7	208	51	3
10/02	16	234	75	2
11/02	6	225	49	6
12/02	18	258	56	6
13/02	11	251	70	5
14/02	16	219	61	3
15/02	20	292	68	4
16/02	9	237	61	1
17/02	24	291	64	5
18/02	13	267	70	5
19/02	4	284	64	12
20/02	2	242	72	3
21/02	1	241	69	6
22/02	13	216	47	4
23/02	2	179	38	4
24/02	7	21	48	2
25/02	8	174	47	1
26/02	12	163	56	4
27/02	7	172	52	2
28/02	12	170	46	3
29/02	5	207	42	7
01/03	4	170	48	8
02/03	17	207	52	2
03/03	10	155	48	3
04/03	3	168	51	3
05/03	9	135	31	4
06/03	5	147	50	5
07/03	10	110	39	1
08/03	7	85	22	2
09/03	3	105	35	3

**Table 2:** Number of patients for triage category from March 10th to April 9th, 2020

Data	#patients in white category of triage	#patients in green category of triage	#patients in yellow category of triage	#patients in red category of triage
10/03	5	83	33	4
11/03	4	84	38	1
12/03	2	92	36	1
13/03	5	66	39	3
14/03	6	81	29	0
15/03	1	40	26	2
16/03	4	56	30	4
17/03	7	62	23	1
18/03	3	66	20	4
19/03	1	63	23	2
20/03	2	68	22	6
21/03	0	49	23	2
22/03	2	47	33	5
23/03	6	55	21	3
24/03	1	56	22	5
25/03	3	54	21	2
26/03	1	47	32	4
27/03	3	54	23	6
28/03	3	53	23	3
29/03	2	32	23	5
30/03	3	49	24	4
31/03	2	43	29	1
01/04	0	47	27	1
02/04	9	45	21	2
03/04	3	59	29	2
04/04	4	52	25	0
05/04	0	45	27	0
06/04	4	63	25	6
07/04	1	44	26	5
08/04	7	60	24	6
09/04	3	56	32	5

Already from a preliminary analysis of the data relating to the numbers of patients for each triage category, it is possible to see a noticeable difference in turnout between the two periods under consideration; access to the ED for mild symptoms (green category of triage) decreased from a medium of 194,43 patients per day to a medium of 57,13 patients per day, which means a 70,61% reduction. Using the EDWIN and NEDOCS overcrowding indexes, the expected result must be consistent with the data presented so far, showing values that will drastically decrease during the second period of time considered in our study.

### The NEDOCS model

The NEDOCS index (National ED Overcrowding Study Index) compiled by Weiss et al. [40] in 2004 is defined as

$$-20+85.8*\left(\frac{TP}{ED Bds}\right)+ 600*\left(\frac{Brdg}{H Bds}\right)+ 13.4*\left(Vent\right)+0.93*\left(Long Admt\right)+5.64*(LBT)$$

where the variables are as follows:

- TP: Total number of patients present in the emergency room
- ED Bds: Total number of beds in the ED
- Brdg: Total number of patients waiting for treatment
- H Bds: Number of accredited hospital beds
- Vent: The number of patients undergoing respiratory care
- Long Admt: Longest wait time (in hours) for patients awaiting treatment
- LBT: Waiting time of the last patient called from the waiting room (door-to-bed)

The number of accredited beds of “San Giovanni di Dio e Ruggi d'Aragona" University Hospital of Salerno is 642, while the total number of beds in the ED is 25. The analysis of other parameters has been carried out day by day, hour by hour.

Pearson correlation coefficient

The Pearson correlation coefficient is a measure of the linear dependence between two random variables (real-valued vectors). Historically, it is the first formal measure of correlation and it is still one of the most widely used measure of relationship. The Pearson correlation coefficient of two variables *x* and *y* is formally defined as the covariance of the two variables divided by the product of their standard deviations (which acts as a normalization factor) and it can be equivalently defined by [41]:

$$r=\frac{\sum \left({x}_{i}-\overline{x }\right)\sum \left({y}_{i}-\overline{y }\right)}{\sqrt{\sum {({x}_{i}-\overline{x })}^{2}} \sqrt{\sum {\left({y}_{i}-\overline{y }\right)}^{2}}}$$

where $\overline{x }=\frac{1}{n}{\sum }_{i=1}^{N}{x}_{i}$ denotes the mean of *x* and $\overline{y }=\frac{1}{n}{\sum }_{i=1}^{N}{y}_{i}$ denotes the mean of *y*.

The coefficient r_xy_ ranges from − 1 to 1 and it is invariant to linear transformations of either variables. The PCC gives an indication on the strength of the linear relationship between the two random variables *x* and *y*. The sign of the correlation coefficient is positive if the variables are directly related and negative if they are inversely related. If r_xy_ = 0, then *x* and *y* are said to be uncorrelated. The closer the value of |r_xy_| is to 1, the stronger the measures closeness to a linear relationship. This is because the association measure reflects the tendency of changes for each pair of corresponding expression levels in the two profiles. The Pearson correlation coefficient measures the similarity of the changes in the expression levels of two profiles. Specifically it measures the strength of the linear relationship between two profiles [42].

## Results

**Fig. 1:**
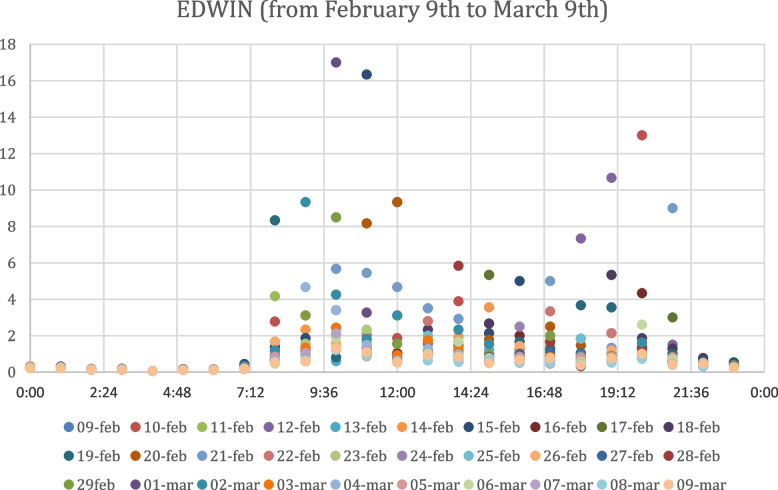
Edwin Index Values from February 9th to March 9th

The values of the EDWIN index obtained after the analysis of the data available from February 9th to March 9th are displayed in Fig. 1, reporting different colours for each of the 30 days of the considered period. In order to better interpret the scatter plot obtained, it is necessary to take into account that an EDWIN score less than 1,5 represents an operational but manageable ED, an EDWIN score between 1,5 and 2 represents a busy ED, and an EDWIN value more than 2 represents an overcrowded ED.

**Fig. 2:**
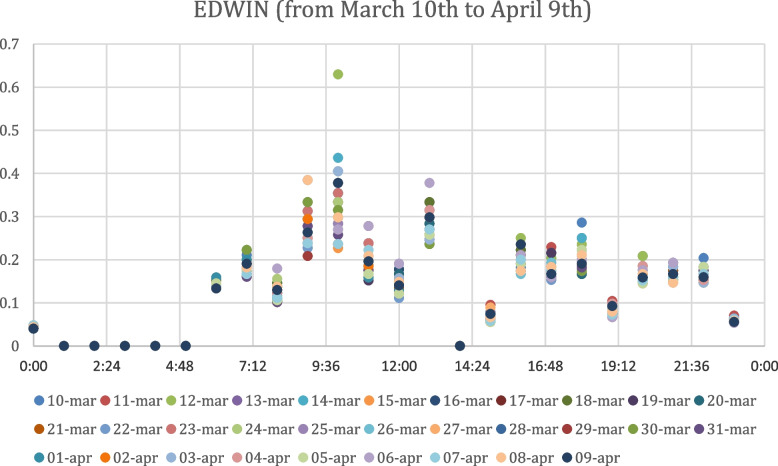
Edwin Index Values from March 10th to April 9th

The values of the EDWIN index obtained after the analysis of the data available from March 10th to April 9th are displayed in Fig. 2, reporting different colours for each of the 31 days of the considered period.

**Fig. 3:**
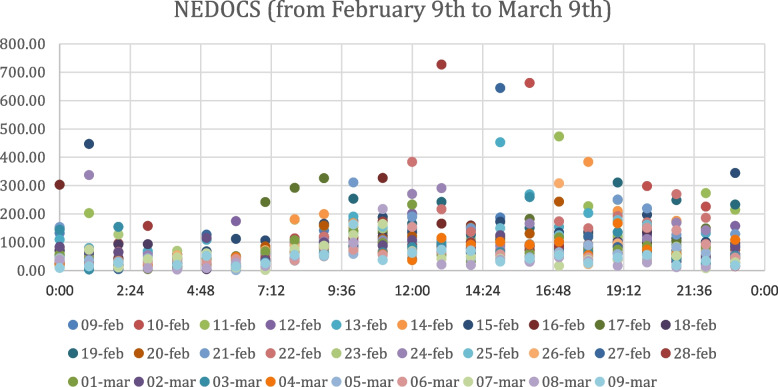
Nedocs Index Values from February 9th to March 9th

**Fig. 4:**
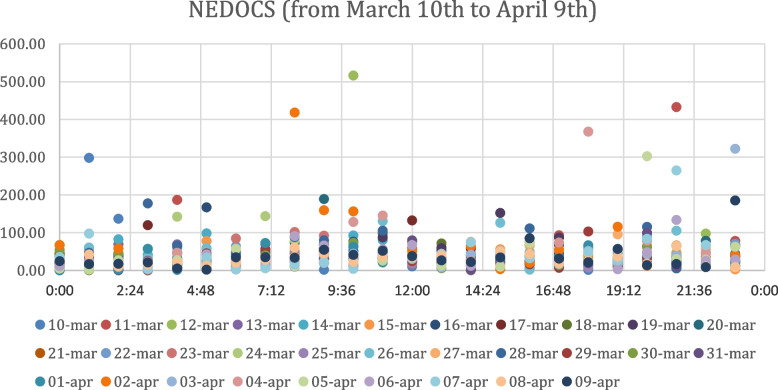
Nedocs Index Values from March 10th to April 9th

The values of the NEDOCS index are given in Fig. 3 and Fig. 4 for the two considered periods, respectively, with distinct colours for each day, as with the EDWIN index. In order to better interpret the scatter plot obtained, it is necessary to take into account that values of the NEDOCS index between 0 and 50 indicate a regular ED condition, values between 50 and 101 suggest busy, values between 101 and 140 indicate overcrowding, values between 141 and 180 indicate extreme overcrowding, and values > 180 imply disaster.

Finally, the Pearson correlation coefficient has been calculated considering as first variable the EDWIN scores obtained hour per hour from 9th February to 9th April, 2020 and as second variable the NEDOCS scores obtained hour per hour from 9th February to 9th April, 2020. The result obtained is shown below:

*r* = 0,317,531.

## Discussion

In this work, an overcrowding measure was implemented extrapolating data from the management software of the "San Giovanni di Dio e Ruggi d'Aragona" University Hospital of Salerno in order to evaluate the effectiveness of EDWIN and NEDOCS indexes. The present examination extended and enhanced a prior study that has been presented at the BECB conference in August 2021. Specifically, in this work we considered a longer period of time, thus improving the accuracy of the evaluation, and we conducted a more accurate analysis on the possible correlation between the two considered indexes, assessing which of them best reproduced the actual condition of the Emergency Department in the University Hospital [36].

It is possible to observe the scatter plots of EDWIN values obtained by the analysis of the ED data from February 9th to March 9th, 2020 in Fig. 1 and the ED data from March 10th to April 9th, 2020 in Fig. 2. As expected, in the second scatter plot the EDWIN index scale shrinks dramatically, with the order of magnitude dropping from 10^1^ to 10^–1^, from a maximum point between 16 and 18 (Fig. 1) to a maximum point between 0,6 and 0,7 (Fig. 2). The evidence that can be demonstrated is that the EDWIN index follows a trend consistent with the situation of the first lockdown period in Italy, defined by extreme limitations imposed by the central government owing to the breakout of the Covid-19 epidemic. In particular, the Ministry of Health advised that in the event of symptoms or doubts, people should stay at home rather than going to the emergency department or doctor's offices; instead, they should call their own family doctor, paediatrician, or doctor on phone [43]. Certainly, during the lockdown, telemedicine consultations were used wherever feasible to prevent entrance to the emergency department, save for clinical or therapeutic purposes. Despite the fact that the EDWIN index scale has shrunk, the 8:00–20:00 time frame remains the most congested, with peak values between 8:00 and 12:00.

We would expect to see a similar trend for NEDOCS index values as well observing the scatter plots of NEDOCS values obtained by the analysis of the ED data from February 9th to March 9th, 2020 in Fig. 3 and the ED data from March 10th to April 9th, 2020 in Fig. 4.; on the contrary, in NEDOCS index charts it isn’t possible to observe the same contraction of the order of magnitude. This phenomenon can be associated with the fact that it is calculated using different parameters than EDWIN index. The order of magnitude of index value doesn’t show a severe contraction, hovering around 10^2^.

Also as regards the analysis of overcrowding by time bands, it is not possible to clearly distinguish the most crowded times from the scatter plots in Fig. 3 and Fig. 4, except for peaks between 08:00 and 12:00 and between 17:00 and 22:00 during the period from March 10th to April 9th. Thanks to the ability of the EDWIN index to reproduce the real context that occurred in hospitals during the period considered, our study is an effective tool to measure the situation in the Emergency Department before and after the Covid period, representing a novelty compared to previous studies. In addition, the ability of this index to predict the status of overcrowding could be used as a support to detect those time periods most touched by crowding and then distribute the necessary medical resources appropriately. Moreover, unlike previous studies, our analysis recorded the state of the ED considering separately for each day the situation in intervals of one hour over a period of two months, increasing the sensitivity of the forecast.

Another result obtained by this study regards the correlation analysis carried out between EDWIN and NEDOCS indexes. As shown in paragraph 3, the value of the correlation coefficient is 0,317: the positive sign means that the variables are directly related but the value of the Pearson coefficient suggests that measures are far from having a linear relationship between each other. This means that, in the same overcrowded circumstance, they could assume different values ​​that do not fully reflect the real observed situation.

Overcrowding in the ED has become an increasingly significant public health problem produced by several factors both internal and external to the hospital facility. Insufficient beds, staff lack are just a few examples of internal deficiencies that could generate this issue [44]. In addition to these as anticipated, external causes such as increasing patient volume or complexity of cases treated, contribute [35]. This is the situation that had to be dealt with in the COVID-19 era where all resources were focused on the treatment of this new disease causing severe effects on the population. Its spread, however, especially in the early months, was not uniform. In some places, there was no overflow but on the contrary, a massive decrease was observed, reaching up to -50% of the admission rate [45]. Kurt et al. [46] in their study highlight and analyze this reduction predicting, in addition, a more critical situation in the near future due to patients with worse prognoses. The fear of contagion, the obligation to limit movements and the blocking of activities in elections have led patients to tolerate slight symptoms that could cause some potentially fatal conditions to be missed in a timely manner, forcing them to present to emergency services with a worse prognosis. Similar studies have also been conducted in Italy, the reference country for this study, demonstrating the danger of this phenomenon [47]. In addition to this, the reduction in accesses could be attributable to a reduction in other seasonal infections due to self-isolation or to a more appropriate use of the ED, limiting accesses for non-relevant pathologies [48]. In fact, for many patients, the emergency department is the place to do several tests together, free of charge and without waiting lists [49].

Now that the most critical situation seems to be over, it is good to analyze what happened in order to learn a lesson. The hospital under our study was not affected in the first months under analysis by a COVID-19 patient flow and, therefore, offers important insights in this area.

In our work, in fact, it addresses the issue of the impact of COVID-19 on ED accesses not by basing it on a simple statistical analysis, but by validating it through a validated methodology well known in the literature as that of indices. Indices, in fact, serve to quantitatively describe a perception of overcrowding by converting a set of data, organizational and clinical, into a single objective and directly comparable number [50]. To this, we add the comparison of two different methodologies (EDWIN and NEDOCS) by concluding with a correlation study between the two results, which is still a poorly covered topic in the literature [51]. The choice of this time interval is due to a desire to understand how this phenomenon changed as soon as the nation and especially the government became aware of an uncontrolled spread of the virus and put in place significant corrective measures, such as lockdown.

From this study, appropriately integrated with the clinical variables of the patients treated, there will be a significant clinical and especially organizational impact. Indeed, it will be possible to put in place internal corrective measures affecting the organization of work or reorganizing staff and especially external ones by improving the health education of users to avoid inappropriate access, as well as an expansion of the prehospital role of primary care and better access to alternative health services.

However, our work is not without limitations. In fact, it is a single-center study that does not allow generalization of the results obtained, based on a limited observation time without including clinical data of treated patients that could offer important discussion points of the phenomenon analyzed.

## Conclusions

The research presented in this study contributes to a better understanding of congestion and the current status of the emergency department. The findings reveal that the two scales, the EDWIN and the NEDOCS, have different outcome variables of ED overcrowding. In particular, knowing a priori the condition of Emergency Departments in Italy during the periods under study, we can better evaluate the capacity of EDWIN and NEDOCS indexes to forecast ED's overcrowding condition. This strategy might be used to identify times of day when the ED is particularly busy and hence deploy the required healthcare resources. Future development of this study could foresee the enlargement of the sample of data to be analyzed in order to make the results more and more accurate; in fact one of the limitations of this study could be the limited period of time considered for data analysis. Besides, it could be interesting comparing the results of the data analysis from dataset belonging to different hospital structures in order to understand the dynamics that led to crowding peaks. From our study, however, we can affirm that EDWIN score represents a more coherent solution to represent ED overcrowding; therefore with respect to the parameters taken into consideration in the evaluation of the NEDOCS score, the number of patients in the emergency room per triage category, the number of physicians on duty, the number of treatment beds and the number of patients in the ED represent the most significant parameter to take into account. Besides, the results obtained by the correlation analysis between EDWIN and NEDOCS scales suggest that there is not a sufficiently significant degree of dependence between them. In conclusion, we can confirm that EDWIN scale demonstrate good discrimination for foreseeing ED overcrowding, which proves the validity of this index as methodologies for overcrowding measurement. *This type of approach could represent a mean for the management of the hospital thanks to which to organize resources and foresee any critical situations on the basis of previous knowledge.*

## Data Availability

The dataset generated and analyzed during the current study is not publicly available due the hospital privacy policies. The hospital structure has given availability to the use of anonymized data for scientific research purposes but not for disclosure. Following a formal request, we obtained anonymized data for research purposes. For this reason, the dataset is available from the corresponding author.

## Abbreviations

ED: Emergency Department
ACEP: American College of Emergency Physicians
DH: Department of Health
LOS: Length Of Stay
EDWIN: Emergency Department Work Index
READI: Real-time Emergency Analysis of Demand Indicators
NEDOCS: National ED Overcrowding Study Index
NEAT: National Emergency Access Target
PCC: Pearson Correlation Coefficient
